# Aroma-Active Compounds in Jinhua Ham Produced With Different Fermentation Periods

**DOI:** 10.3390/molecules191119097

**Published:** 2014-11-19

**Authors:** Xiao-Sheng Liu, Jian-Bin Liu, Zheng-Mao Yang, Huan-Lu Song, Ye Liu, Ting-Ting Zou

**Affiliations:** Laboratory of Molecular Sensory Science, College of Food and Chemical Engineering, Beijing Technology and Business University, Beijing 100048, China; E-Mails: liuxaosheng@126.com (X.-S.L.); localcast@163.com (J.-B.L.); yangzhengmao86@163.com (Z.-M.Y.); liuye@th.btbu.edu.cn (Y.L.); zoutingting@th.btbu.edu.cn (T.-T.Z.)

**Keywords:** Jinhua ham, aroma-active compounds, GC-O-MS, DHS, SAFE

## Abstract

The aroma-active compounds in Jinhua ham processed and stored for 9, 12, 15 and 18 months were extracted by dynamic headspace sampling (DHS) and solvent-assisted flavor evaporation (SAFE) and analyzed by gas chromatography-olfactometry-mass spectrometry (GC-O-MS). In GC-O-MS, volatile compounds were identified based on their mass spectrum, linear retention index (LRI), odor properties, or reference compound comparisons. The results showed that a total number of 81 aroma-active compounds were identified by GC-O-MS. Among them, acids (such as acetic acid, butanoic acid and 3-methylbutanoic acid), saturated aldehydes (such as hexanal, heptanal, octanal and 3-methylbutanal), benzene derivatives (such as benzeneacetic acid), ester and lactone (such as γ-nonalactone and γ-decalactone) were identified as critical compounds in Jinhua ham aroma. The results also indicated that the type and content of the odorants increased significantly with the duration of the fermentation period.

## 1. Introduction

Dry-cured ham is generally classified based on the origin. In particular, the three main forms from southern China, southern or central Europe and the southeastern United States, have many differences in their sensory properties [1]. Jinhua ham, Parma ham, Iberian ham and the American ham are their best known representatives [2,3,4,5]. In China, Jinhua ham, along with “Xuanwei ham” and “Rugao ham”, are well known as the “three hams”. The traditional processing technology for making Jinhua ham is composed of multiple steps, including raw material selection, salting, soaking and washing, sun drying and shaping, fermentation, ripening, post-ripening, grading and storage [6]. The unique flavor of Jinhua ham is appreciated by consumers all over the world. Nowadays, ham quality is graded by its aroma intensity and persistence on the bamboo stick, but different processing technologies can make a great difference in the flavor quality of ham. Therefore, the control of ham flavor formation during processing is very important for ham grading, so comprehensive research of Jinhua ham flavor is crucial for better ham quality and the establishment of a national traditional meat products standard.

In the fermentation process, relevant chemical and biological reactions take place in the muscle of Jinhua ham, such as lipid degradation and oxidation, Maillard reactions, Strecker degradation,* etc.*, resulting in the special ham flavor [7]. Many exploration methods and technologies have been used to analyze the odorants in Jinhua ham, including dynamic headspace sampling (DHS) [8], purge-and-trap (P&T) [9], solid phase microextraction (SPME) [10], but to our knowledge, there are few studies on the identification of variations of key odorants of Jinhua ham at different fermentation stages, compared to that of Western dry-cured hams such as Parma and Iberian ham [11,12,13,14,15,16].

The objective of this study was to identify and characterize the aroma-active compounds of Jinhua ham under different processing times and operation conditions by gas chromatography-olfactometry-mass spectrometry (GC-O-MS), aided by both dynamic headspace dilution analysis (DHDA) and aroma extract dilution analysis (AEDA) techniques.

## 2. Results and Discussion

### 2.1. Aroma-Active Compounds

**Figure 1:**
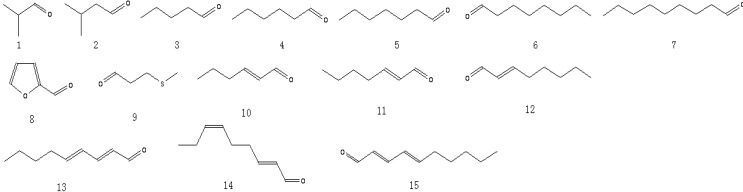
Structures of the saturated and unsaturated aldehydes in Jinhua ham.

**Figure 2:**
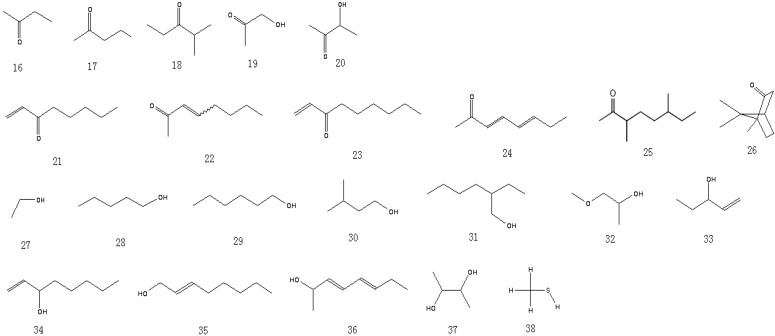
Structures of the ketones and alcohols in Jinhua ham.

**Figure 3:**
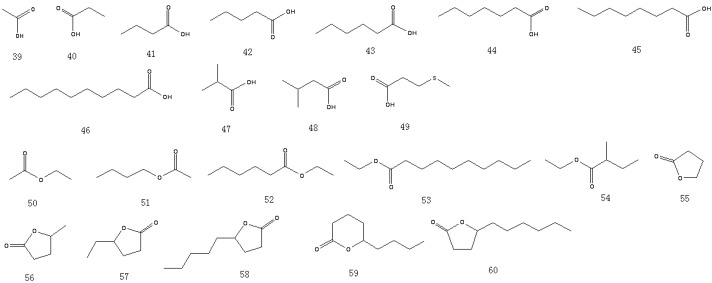
Structures of the acids, esters and lactones in Jinhua ham.

**Figure 4:**
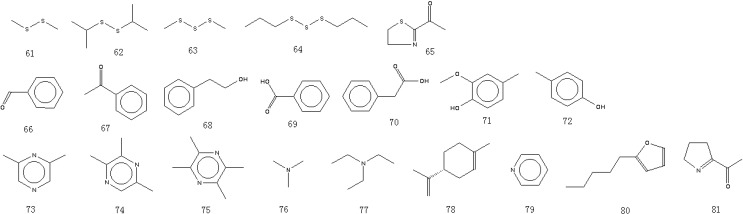
Structures of the sulfides, benzene series compounds, pyrazines and other compounds in Jinhua ham.

A total of 81 aroma-active compounds of Jinhua ham in different processing time were identified by DHS-GC-O-MS and SAFE-GC-O-MS. The compounds included 15 saturated and unsaturated aldehydes (Figure 1), 11 ketones (Figure 2), 12 alcohols (Figure 2), 11 acids (Figure 3), 11 esters and lactones (Figure 3), five sulfides (Figure 4), seven benzene derivatives compounds (Figure 4), three pyrazines and six others (Figure 4). 

### 2.2. Key Aroma-Active Compounds by Dynamic Headspace Dilution Analysis (DHDA)

Sixty-seven compounds were identified as odorants by DHS-GC-O-MS (Table 1) and eight compounds remained unknown. Among the identified compounds, 3-methylbutanoic acid (odor: sour and sweaty), 2-acetyl-1-pyrroline (odor: popcorn), trimethylamine (odor: fishy) and γ-nonalactone (odor: peachy and sweet) had average FD factors over one hundred (where Average FD factor = Sum of the FD factors in one compound of 18, 15, 12 and 9 months ham/4). Seven other identified compounds: acetic acid (odor: sour), hexanal (odor: cut-grass), 2,6-dimethylpyrazine (odor: toast and nutty), butanoic acid (odor: cheesy), methional (odor: cooked potato), γ-decalactone (odor: peachy and burnt sugar) and 1-nonen-3-one (odor: mushroom) had average FD factors ≥ 50.

**Table 1:** Aroma-active compounds in Jinhua ham by DHS.

Nr ^a^	Compound Name ^b^	R.I. ^c^		Identification ^d^	Odor Property ^e^	FD ^f^			
		R.I.(DB-5ms)	R.I.(DB-WAX)			18	15	12	9 ^c^
38	methanthiol	-	627	RI,O	rotten egg	25	125	1	-
76	trimethylamine	503	848	RI,O,MS	fishy	125	125	125	25
50	ethyl acetate	610	877	RI,O,MS,STD	fruity/sweet	25	25	1	-
2	3-methylbutanal	638	927	RI,O,MS	chocolate/malty	25	125	5	1
27	ethanol	-	941	RI,O,MS,STD	alcohol	1	1	-	-
77	triethylamine	677	970	RI,O,MS	fishy	25	25	-	-
17	2-pentanone	-	971	RI,O,MS	fruity	5	25	-	-
3	pentanal	700	984	RI,O,MS	fermented/yoghourt	25	5	5	-
54	2-methyl-butanoic acid ethyl ester	850	1051	RI,O,MS	fruity/sweet	25	5	5	1
51	acetic acid butyl ester	812	1059	RI,O	green/fruity	5	5	-	-
18	2-methyl-3-pentanone	-	1068	RI,O,MS	mint	25	5	-	-
61	disulfide, dimethyl	750	1079	RI,O,MS	cooked cabbage/onion	25	25	1	1
4	hexanal	803	1094	RI,O,MS,STD	cut-grass	125	125	25	5
32	1-methoxy-2-propanol	-	1137	RI,O,MS	plastic	1	1	1	1
33	1-penten-3-ol	688	1164	RI,O,MS,STD	buttery/grassy/green	5	1	-	-
	unknown	-	1179	RI,O,MS	popcorn	5	25	-	-
5	heptanal	905	1183	RI,O,MS	oily/green	25	5	1	-
78	D-limonene	1028	1191	RI,O,MS,STD	sweet /orange	5	25	5	-
79	pyridine	757	1193	RI,O,MS	spicy	1	1	1	1
	unknown	-	1199	O	cooked potato	25	125	1	-
30	3-methyl-1-butanol	-	1201	RI,O,MS	fermented/oily/fruity	5	-	-	-
80	2-pentylfuran	995	1231	RI,O,MS,STD	fruity/green	5	5	-	-
52	hexanoic acid ethyl ester	1000	1232	RI,O,MS	fruity/apple	-	-	5	1
28	1-pentanol	760	1250	RI,O,MS	green	1	5	-	1
10	(E)-2-hexenal	855	-	RI,O,MS	green/fatty	5	5	-	-
	unknown	-	1270	O	popcorn	125	125	5	-
6	octanal	1009	1287	RI,O,MS	fatty	5	5	1	1
22	3-octen-2-one	1045	1285	RI,O,MS	fatty/nutty/spicy	25	125	-	-
20	3-hydroxy-2-butanone	722	1286	RI,O,MS	buttery/green	5	5	1	-
21	1-octen-3-one	980	1297	RI,O,MS,STD	mushroom	125	125	5	5
19	1-hydroxy-2-propanone	680	1307	RI,O,MS	nutty/bitter	-	-	1	5
11	(E)-2-heptenal	961	1317	RI,O,MS,STD	fatty/fruity	25	25	-	-
81	2-acetyl-1-pyrroline	912	1339	RI,O,STD	popcorn	625	125	125	25
	unknown	-	1345	O	fishy	25	25	-	-
73	2,6-dimethylpyrazine	919	1337	RI,O,MS,STD	toast/nutty	125	125	25	5
63	dimethyl trisulfide	-	1380	RI,O,MS	garlic/cooked cabbage	125	25	5	-
74	trimethylpyrazine	1006	1395	RI,O,MS,STD	nutty/chocolate	25	5	1	1
23	1-nonen-3-one	-	1404	RI,O,MS	mushroom	25	5	1	-
7	nonanal	1102	1408	RI,O,MS	green/fatty/soapy	25	1	5	-
36	3,5-octadien-2-ol	-	1408	RI,O,MS	green	1	-	-	-
12	(E)-2-octenal	1064	1425	RI,O,MS	fatty	25	5	-	-
75	tetramethylpyrazine	1084	-	RI,O,MS,STD	nutty	1	-	-	1
34	1-octen-3-ol	986	1445	RI,O,MS	mushroom	5	25	25	25
39	acetic acid	658	1446	RI,O,MS,STD	sour	125	125	25	25
9	methional	906	1450	RI,O	cooked potato	125	125	25	-
8	furfural	-	1453	RI,O,STD	sweet popcorn/wood	125	25	-	-
26	camphor	1145	1503	RI,O,MS	camphor	5	1	1	-
47	2-methylpropanoic acid	828	1550	RI,O,MS	sock/stinky	25	125	1	1
66	benzaldehyde	969	1515	RI,O,MS	almond	5	5	-	-
24	(E,E)-3,5-octadien-2-one	1091	1520	RI,O,MS	fatty	5	-	1	-
40	propanoic acid	718	1556	RI,O,MS	sour	25	25	-	-
	unknown	-	1559	O	chocolate	25	1	1	-
37	2,3-butanediol	783	1581	RI,O,MS	fruity/creamy/oily	-	-	5	25
14	(E,Z)-2,6-nonadienal	-	1586	RI,O	cucumber	1	1	1	-
35	(E)-2-octen-1-ol	-	1607	RI,O,MS	mushroom	-	5	-	-
55	butyrolactone	950	1613	RI,O,MS	hay	5	25	1	-
	unknown	-	1617	O	rancid/fishy	125	125	-	-
41	butanoic acid	821	1630	RI,O,MS	cheesy	125	125	25	5
56	γ-pentalactone	-	1632	RI,O,MS	creamy	25	1	-	-
53	decanoic acid, ethyl ester	-	1637	RI,O,MS	fruity	5	1	-	1
67	acetophenone	1078	1612	RI,O,MS	flower/sweet	-	-	1	1
48	3-methylbutanoic acid	876	1667	RI,O,MS	sour/sweat	625	625	625	125
13	(E,E)-2,4-nonadienal	1155	1703	RI,O,MS	fatty/fried	5	1	-	-
57	γ-hexanolactone	1062	1705	RI,O,MS	hay/sweet	25	1	-	-
42	pentanoic acid	911	1714	RI,O,MS	meat/rancid	125	25	1	1
65	2-acetyl-2-thiazoline	-	1759	RI,O	popcorn	1	5	5	-
15	(E,E)-2,4-decadienal	-	1812	RI,O,MS,STD	fatty/fried	5	-	1	-
43	hexanoic acid	997	1833	RI,O,MS	sour/rancid	5	1	-	-
71	4-methylguaiacol	-	1938	RI,O	mushroom/smoke	25	5	-	-
58	γ-nonalactone	1124	2023	RI,O	peachy/sweet	125	125	125	25
72	p-cresol	-	2031	RI,O	fecal	125	25	25	1
59	δ-nonalactone	1363	2035	RI,O,MS	coconut/creamy/sweet	1	25	5	25
60	γ-decalactone	1370	2150	RI,O	peachy	125	125	25	-
	unknown	-	2178	O	medicine	25	25	5	-
	unknown	-	2185	O	burnt sugar	125	125	-	-

Notes: **^a^** Nr was corresponded to the compound number in Figure 1, Figure 2, Figure 3 and Figure 4; **^b^** Compound name: The compounds were identified in Jinhua ham; **^c^** Linear retention index (LRI) for the odorants in DB-5ms and DB-WAX column; **^d^** Method of odorant identification included RI,O, MS and STD which represented linear retention index, odor property, mass spectrum and authentic standards verification in GC-MS; **^e^** Odor descriptors are those used by all assessors, with most frequently used terms cited first; **^f^** Nitrogen stream purging at 70 mL/min for 50, 10, 2, 0.4 min respectively, the FD factors were 1, 5, 25,125 respectively; purging at 14 mL/min for 0.4 min, the FD factor was 625.

**Figure 5:**
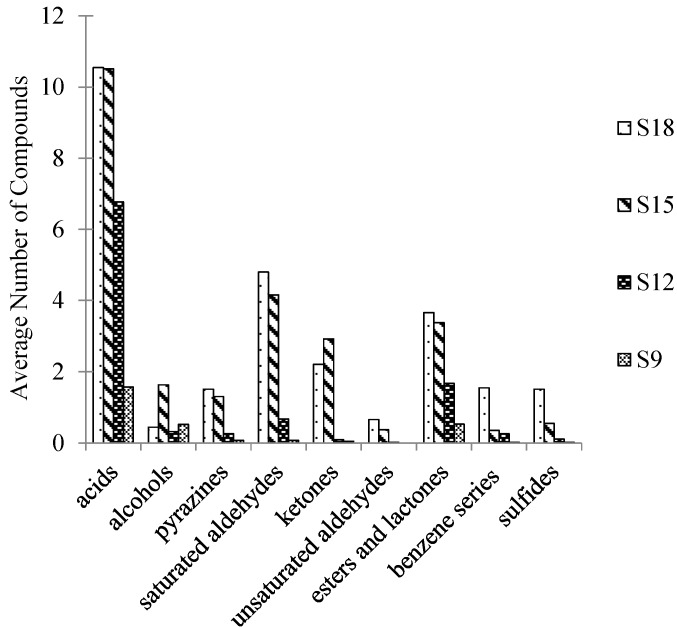
The category of compounds in Jinhua ham by DHS. D9, D12, D15 and D18 referred to Jinhua ham processed for 9, 12, 15 and 18 months and application of DHS for aroma extraction of sample, respectively.

In Table 2, combined with the GC-O results, 13 critical compounds were selected to calculate their respective odor activity value (OAV). Generally, the compound contributed to the whole flavor profile if its OAV ≥ 1. Table 2 showed that all the five saturated aldehydes, three acids, one alcohol, one sulfide and three other compounds were identified as the key compounds that contribute to the overall aroma of Jinhua ham. Figure 5 shows the category of compounds in ham by DHS. It was obvious that, along with the increase of the fermentation time, the kind and content of the odorants increased, especially in the categories of acid, saturated aldehyde, ester and lactone. Among them, odorants such as acetic acid, butanoic acid and 3-methylbutanoic acid made contributions of sour, sock and cheesy notes to the overall aroma profile of Jinhua ham. Therefore as aldehydes like hexanal, heptanal, octanal and 3-methylbutanal made odor impacts of green, fatty and chocolate-like notes, and ester and lactones such as γ-nonalactone and γ-decalactone contributed fruity, sweet and creamy odors.

**Table 2:** OAVs of predominant odorants in Jinhua hams by DHS.

Nr ^a^	Compound Name	Odor Property	Threshold (ppb in Water)	18-inner	18-inner	15-inner	15-inner	12-inner	12-inner	9-inner	9-inner
				Concn (ppb)	OAV	Concn (ppb)	OAV	Concn (ppb)	OAV	Concn (ppb)	OAV
76	trimethylamine	fishy	0.7	2280 ± 234	3256	730 ± 36	1043	179 ± 35	256	327 ± 22	467
2	3-methylbutanal	chocolate/malty	0.2	2892 ± 373	14,458	1413 ± 127	7066	71 ± 28	356	1121 ± 65	5607
3	pentanal	fermented/yoghourt	25	-	-	526 ± 33	21	358 ± 21	14	-	-
61	dimethyl disulfide	cooked cabbage/onion	6	1358 ± 279	226	736 ± 61	123	725 ± 63	121	41 ± 9	7
4	hexanal	cut-grass	4.5	2719 ± 252	604	1414 ± 221	314	1896 ± 98	421	152 ± 12	34
5	heptanal	oily/green	3	297 ± 33	100	541 ± 26	180	128 ± 12	43	9 ± 2	3
78	D-limonene	sweet/orange-like	10	248 ± 31	25	-	-	223 ± 49	22	323 ± 33	32
80	2-pentylfuran	fruity/green	6	61 ± 27	10	529 ± 41	88	35 ± 10	6	114 ± 27	19
6	octanal	fatty	0.7	148 ± 46	211	421 ± 68	602	92 ± 15	132	5 ± 1	7
34	1-octen-3-ol	mushroom	1	243 ± 37	243	-	-	-	-	5 ± 1	5
39	acetic acid	sour	22,000	52,796 ± 1673	2	21,837 ± 751	1	-	-	-	-
41	butanoic acid	cheesy	240	3167 ± 121	13	1887 ± 189	8	1864 ± 149	8	-	-
48	3-methylbutanoic acid	sour/sweaty	120	2842 ± 336	24	1533 ± 267	13	2601 ± 259	22	-	-

Note: **^a^** Nr was corresponded to the compound number in Figure 1, Figure 2, Figure 3 and Figure 4.

**Table 3:** Aroma-active compounds in Jinhua ham by SAFE.

Nr ^a^	Compound Name ^b^	R.I. ^c^		Identification ^d^	Odor Property ^e^	Log_3_FD ^f^			
		R.I.(DB-5ms)	R.I.(DB-WAX)			18-inner	15-inner	12-inner	9-inner ^c^
1	methylpropanal	540	867	RI,O,MS	green/floral	-	-	<1	<1
50	ethyl acetate	605	880	RI,O,MS,STD	fruity/sweet	-	2	-	2
16	2-butanone	603	900	RI,O,MS	green	2	-	-	-
27	ethanol	-	930	RI,O,MS,STD	alcohol	-	-	1	1
	unknown	-	-	-	floral/sweet	1	1	-	3
54	2-methyl-butanoic acid ethyl ester	855	1049	RI,O,MS	fruity/sweet	2	-	-	2
61	dimethyl disulfide	750	1079	RI,O,MS	cooked cabbage/onion	1	4	<1	<1
4	hexanal	790	1084	RI,O,MS,STD	cut-grass	4	3	2	1
80	2-pentylfuran	990	1240	RI,O,MS,STD	fruity/green	1	3	2	<1
52	hexanoic acid ethyl ester	999	1232	RI,O,MS	fruity/apple	-	<1	-	<1
62	isopropyl disulfide	1018	-	RI,O,MS	sulfurous	2	1	1	-
20	3-hydroxy-2-butanone	720	1286	RI,O,MS	buttery/green	1	-	-	<1
6	octanal	1001	1280	RI,O,MS	fatty	3	2	2	<1
21	1-octen-3-one	-	1289	RI,O,STD	mushroom	3	3	3	2
73	2,6-dimethylpyrazine	922	1308	RI,O,MS,STD	toast/nutty	3	4	2	1
29	1-hexanol	869	1360	RI,O,MS	leafy/green	1	1	-	-
63	dimethyl trisulfide	-	1377	RI,O,MS	garlic/cooked cabbage	4	1	4	-
7	nonanal	1102	1385	RI,O,MS	green/fatty/soapy	2	2	2	1
39	acetic acid	625	1450	RI,O,MS,STD	sour	4	2	3	3
9	methional	-	1455	RI,O	cooked potato	4	3	5	3
31	2-ethyl-1-hexanol	-	1487	RI,O,MS	green	1	1	-	-
40	propanoic acid	668	1523	RI,O,MS	sour	1	<1	1	1
64	dipropyl trisulfide	1104	1536	RI,O,MS	garlic/onion/penetrating	<1	2	-	-
37	2,3-butanediol	800	1583	RI,O,MS	fruity/creamy/oily	<1	1	2	1
47	2-methyl-propanoic acid	790	1501	RI,O,MS	sock/stinky	2	-	-	1
41	butanoic acid	815	1621	RI,O,MS	cheesy	4	4	4	4
48	3-methyl-butanoic acid	848	1664	RI,O,MS	sour/sweat	5	5	5	4
42	pentanoic acid	933	1719	RI,O,MS	meaty/rancid	2	3	1	1
15	(E,E)-2,4-decadienal	-	1812	RI,O,MS,STD	fatty/fried	4	2	2	-
43	hexanoic acid	997	1856	RI,O,MS	sour/rancid	3	2	2	-
68	phenylethyl alcohol	-	1895	RI,O,MS	rosy	1	-	1	-
44	heptanoic acid	1076	1932	RI,O,MS	sour	1	1	-	-
58	γ-nonalactone	-	2035	RI,O,MS	peachy/sweet	5	5	5	2
45	octanoic Acid	1186	2040	RI,O,MS	sour	1	1	2	1
72	p-cresol	1084	2056	RI,O,MS	fecal	4	2	3	3
25	3,6-dimethyl-octan-2-one	-	2078	RI,O,MS	fatty	1	-	-	-
60	γ-decalactone	-	2150	RI,O	peachy	5	5	4	3
46	decanoic acid	1382	2259	RI,O,MS	smoky/acid	1	-	2	<1
49	3-(methylthio)-propanoic acid	-	2293	RI,O,MS	oily/acid	2	1	-	-
69	benzoic acid	-	2453	RI,O,MS	benzoin/balsam	1	2	-	-
70	benzeneacetic acid	-	2562	RI,O,MS	rosy	5	5	4	2

Notes^: **a**^ Nr was corresponded to the compound number in Figure 1, Figure 2, Figure 3 and Figure 4.; **^b^** Compound name: The compounds were identified in Jinhua ham; **^c^** Linear retention index (LRI) for the odorants in DB-5ms and DB-WAX column; **^d^** Method of odorant identification included RI,O, MS and STD which represented linear retention index, odor property, mass spectrum and authentic standards verification in GC-MS; **^e^** Odor descriptors are those used by all assessors, with most frequently used terms cited first; **^f^** Serial dilutions (1:3, 1:9, 1:27 and so on) in mixed solution of redistilled ether and n- pentane (v:v = 2:1), the Log_3_FD were 1, 2, 3 and so on respectively.

**Table 4:** OAVs of predominant odorants in Jinhua hams by SAFE.

Nr ^a^	Compound Name	Odor Property	Threshold (ppb in Water)	18-inner Concn (ppb)	18-inner OAV	15-inner Concn (ppb)	15-inner OAV	12-inner Concn (ppb)	12-inner OAV	9-inner Concn (ppb)	9-inner OAV
4	hexanal	cut-grass	4.5	986 ± 78	219	122 ± 9	27	112 ± 12	25	-	-
6	octanal	fatty	0.7	264 ± 19	377	48 ± 6	68	92 ± 9	131	-	-
73	2,6-dimethylpyrazine	toast/nutty	200	206 ± 11	1	32 ± 6	0	-	-	-	-
7	nonanal	green/fatty/soapy	1	439 ± 12	439	261 ± 20	261	497 ± 26	497	53 ± 3	53
41	butanoic acid	cheesy	240	2437 ± 121	10	978 ± 34	4	736 ± 31	3	228 ± 13	1
48	3-methylbutanoic acid	sour/sweaty	120	8783 ± 362	73	6199 ± 531	52	466 ± 21	4	1456 ± 98	12
15	(E,E)-2,4-decadienal	fatty/fried	0.07	489 ± 23	6980	306 ± 12	4371	10,971 ± 512	768	-	-
58	γ-nonalactone	peachy/sweet	30	493 ± 11	16	-	-	33 ± 7	1	-	-
72	p-cresol	fecal	55	221 ± 25	4	127 ± 23	2	77 ± 11	1	-	-

Note: **^a^** Nr was corresponded to the compound number in Figure 1, Figure 2, Figure 3 and Figure 4.

### 2.3. Key Aroma-Active Compounds by Solvent-Assisted Flavor Evaporation (SAFE) and Aroma Extract Dilution Analysis (AEDA)

As a rapid and effective method, SAFE maintains the advantages of solvent extraction and avoids the disadvantage of SDE, such as high temperature that can contribute to whole odor changes [17]. The odorants receiving tube was kept under a low temperature (−196 °C) and a high vacuum (10^−4^ Torr) which could increase the capability of trapping of volatiles.

Forty compounds were identified as odorants by SAFE-GC-O-MS (Table 3). The odorants were of middle and high molecular weight. By GC-O, five compounds: 3-methylbutanoic acid (odor: sour and sweaty), γ-nonalactone (odor: peachy and sweet), γ-decalactone (odor: peachy), butanoic acid (odor: cheesy) and benzeneacetic acid (odor: rosy) had the average log_3_FD factor ≥ 4 (Average log_3_FD factor = The sum of the log_3_FD factors in one compound of 18, 15, 12 and 9 months ham/4 and log_3_FD factor < 1 was equal to 0.5). With the development of the degree of fermentation (increase in fermentation time), the main aroma components fell into two odor classes. One was cheesy or sour with the smell of fermentation and the other was sweet, fruity with a pleasurable smell. The following eight identified compounds had the average log_3_FD factor ≥ 2: methional (odor: cooked potato), acetic acid (odor: sour), *p*-cresol (odor: fecal), 1-octen-3-one (odor: mushroom), hexanal (odor: cut-grass), 2,6-dimethylpyrazine (odor: toast and nutty), dimethyl trisulfide (odor: garlic and cooked cabbage) and (*E,E*)-2,4-decadienal (odor: fatty and fried). These compounds enhanced the complexity of ham aroma.

**Figure 6:**
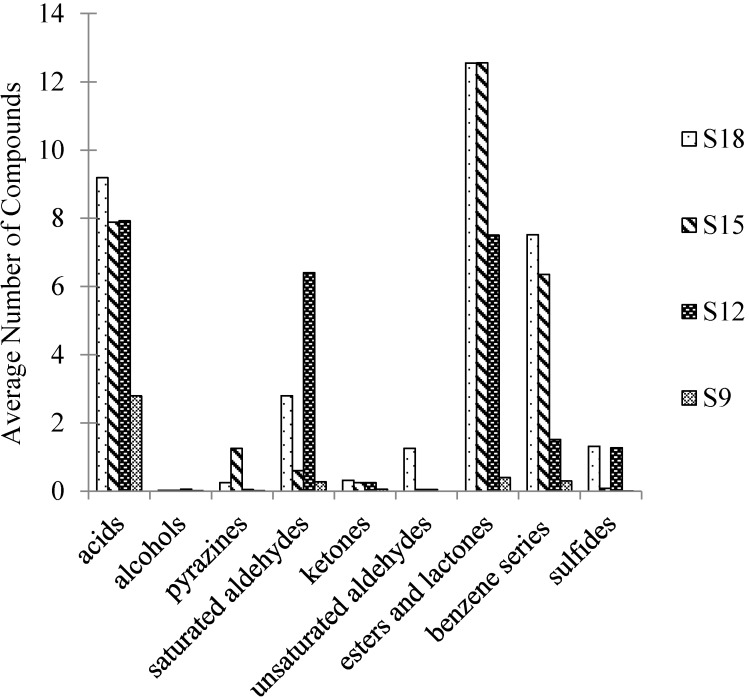
The categories of compounds in Jinhua ham by SAFE. S9, S12, S15 and S18 referred to Jinhua ham processed for 9, 12, 15 and 18 months and application of SAFE was for aroma extraction of samples, respectively.

In Table 4, based on the results by GC-O, nine critical compounds, which include three saturated aldehydes, two acids, one unsaturated aldehyde, one ester and lactone, one benzene series compound and one pyrazine, were selected and their odor activity value (OAV) calculated. However, the OAV conclusion had a limitation due to the fact that the odor thresholds of some compounds in water and ham might be different. The identification of some critical odorants, such as 2-acetyl-1-pyrroline (odor: popcorn) for example, was carried out by comparing the RI and odor quality with a standard substance, because its concentration was too low to be detected by GC-MS. Figure 6 shows the categories of compounds in Jinhua ham by SAFE. The odorants categories were similar to the results by DHS. With the increase of the fermentation time, the type and content of the odorants increased, they were relatively high molecular aroma compounds such as γ-nonalactone, γ-decalactone and benzeneacetic acid, with fruity, creamy and rosy odor properties. Another aspect of the aroma compounds in the AFE extract was that acids and saturated aldehydes were predominant.

The volatile profile of Jinhua hams in different fermentation periods is shown in Figure 5 and Figure 6, which indicate obvious changes during the fermentation period. This is due to a high content of glycerides in the Jinhua ham that could be hydrolyzed with the endogenous enzymes in the fermentation period. Saturated or unsaturated aldehydes have long been reported as the important contributors of the aroma of hams [5,10,18]. Among them, the branched-chain saturated aldehydes such as methylpropanal and 3-methylbutanal in the ham were originated from Strecker degradation of valine and leucine, while the linear chain aldehydes and unsaturated aldehydes were generated from the degradation of long-chain fatty acids [10,18]. Triglycerides and phospholipids could degrade to many free fatty acids, and then degrade further to a considerable amount of linear and branched chain small molecular acids [19]. The branched-chain fatty acids such as propanoic acid, 2-methylpropanoic acid, butanoic acid and 3-methylbutanoic acid were also reported to originate from the oxidation of the corresponding aldehydes, e.g., methylpropanal, 3-methylbutanal,* etc.* [20]. These carboxylic acids were identified as key aroma-active compounds for the mature hams while few of them were identified in the raw ham volatiles [21]. The formation of alcohols from the reduction of aldehydes or ketones was also reported [22]. In addition, ketenes and hydroxyl ketones were the main ketones found in the Jinhua ham. It is well known that 3-hydroxy-2-butanone and 1-hydroxy-2-propanone could originate from glycogen degradation via Maillard reactions [23]. The other 12 alcohols and four ketenes identified in this study were very common in fungal metabolism [24,25]. Acids and alcohols are the key precursors of esters. The fruity/sweet odor component ethyl acetate, 2-methylbutanoic acid ethyl ester, hexanoic acid ethyl ester and decanoic acid ethyl ester come from the esterification of ethanol with acetic acid, 2-methylbutanoic acid, hexanoic acid and decanoic acid, respectively. The milk/peach-like lactones which are the key aroma-active compounds in the odor of Jinhua ham, could be formed from the auto-oxidation of short-chain fatty acids [8].

**Figure 7:**
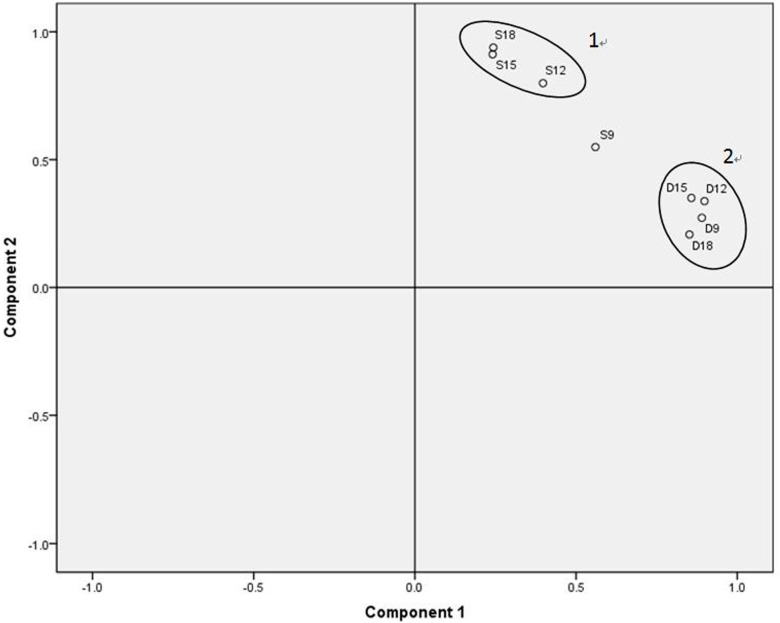
Principal components loadings for different pretreatment methods. D9, D12, D15 and D18 refer to Jinhua ham processed for 9, 12, 15 and 18 months and application of DHS for aroma extraction of samples, respectively. S9, S12, S15 and S18 refer to Jinhua ham processed for 9, 12, 15 and 18 months and application of SAFE for aroma extraction of samples, respectively.

Based on the quantitative data of the volatiles, clustering analysis was done to clearly show the aggregative relationship between the samples. As shown in Figure 7, D9, D12, D15 and D18 referred to the Jinhua ham processed for 9, 12, 15 and 18 months and application of DHS for aroma extraction, while S9, S12, S15 and S18 were referred to the SAFE for sampling. As illustrated by Figure 7, hams with different maturation time were distinguished clearly on the axis. The first two principal components (PC1 and PC2) accounted for 83% of the variance in the data set. The circle 1 in Figure 7 shows that the overall aroma of Jinhua hams tends to be stable as the ripening time reaches to 12 months. In contrast, circle 2 shown that odorants in the headspace of Jinhua ham was almost similar after 9 months of storage. It was reported that the content of free amino acids in the Iberian ham remained almost steady after a 230 days drying stage [16]. Hinrichsen* et al.* also found that the sensory properties of Parma ham showed minor changes after 365 days of storage [26]. 

## 3. Experimental Section

### 3.1. Materials and Chemicals

Jinhua ham samples were supplied by Jinzi Ham Co. Ltd. (Jinhua, Zhejiang Province, China) and the processing time of the hams were 9, 12, 15 and 18 months. Jinhua hams were processed by the traditional ways of raw material selection, salting, soaking and washing, sun drying and shaping, fermentation, ripening, post-ripening, grading and storage. *n*-Alkanes (C_7_–C_22_) and the internal standard (2-methyl-3-heptanone, chromatographic reagent) were obtained from Sigma-Aldrich Co. Ltd. (St. Louis, MO, USA), authentic standards of odorants were also obtained from Sigma–Aldrich Co. Ltd. and Beijing Huihai Scientific Instruments Co., Ltd. (Beijing, China).

### 3.2. Preparation of Jinhua Ham Samples

The biceps muscles parts of the hams were cut into small pieces (approximately 0.2 cm^3^) and frozen in a freezer (control, −80 °C). The frozen samples were used for odorant extraction by dynamic headspace sampling (DHS) and solvent-assisted flavor evaporation (SAFE).

### 3.3. The Extraction of Aroma Compounds by Dynamic Headspace Sampling (DHS)

Dynamic headspace sampling (DHS) was done using a dynamic headspace sampling vessel (150 mL, Kimble Glass, Beijing, China). Volatile compounds of the sample headspace were trapped onto a Tenax TA tube, which was placed onto the vessel. The Tenax tube was then dry purged for 20 min (TD controller, Gerstel, Mülheim an der Ruhr, Germany) to remove moisture for aroma analysis. Aroma compounds from Tenax trap were thermally desorbed at 280 °C using a TDSA2 system (Gerstel) into a cryo-cooled (−150 °C) CIS (the cold injection system) inlet (Gerstel). Injection was splitless (inlet heating rate of 12 °C/min to 260 °C).

### 3.4. Dynamic Headspace Dilution Analysis (DHDA)

Small pieces of hams (1 g) and 2-methyl-3-heptanone (500 ng/g) added as the internal standard were put into the dynamic headspace sampling vessel. After equilibrating 30 min at 50 °C (water-bath circulation), the sample was purged with a nitrogen stream at a flow-rate of 100 mL/min for 60, 12.5, 2.5 or 0.5 min, the FD factors were 1, 5, 25, 125 respectively. Compounds with higher FD are considered to be more critical.

### 3.5. The Extraction of Aroma Compounds by Solvent-Assisted Flavor Evaporation (SAFE)

Small pieces of ham (50 g) and 2-methyl-3-heptanone added as the internal standard (500 ng/g) were mixed in a Soxhlet extraction apparatus (Kimble Glass) , Redistilled ether and *n*-pentane (v:v = 2:1, 120 mL) was used as a solvent in the round bottomed flask (250 mL) at 40 °C in water bath. Soxhlet extraction lasted for 5 h and then applied the solvent-phase extracts into a SAFE apparatus (Deutsche Forschungsanstalt für Lebensmittelchemie, Freising, Germany). The apparatus was made up of vacuum pump, receiving tube and waste tube and it was processed under the low temperature (liquid nitrogen, −196 °C) and at a high vacuum (10^−4^ Torr). Solvent-assisted flavor evaporation was conducted for 1 h to trap the odorants. After the above process, the SAFE extracts were dried with anhydrous sodium sulfate and then frozen at −18 °C for 12 h for removal of moisture. The volume of SAFE extracts was reduced to 0.5 mL under a gentle stream of nitrogen (99.995% purity). The concentrate was stored at in freezer (control, −80 °C) for further analysis.

### 3.6. Aroma Extract Dilution Analysis (AEDA)

Serial dilutions were prepared from the initial SAFE extracts (0.5 mL) in the ratio of 1:3 in diethyl ether. Aliquots were then analyzed by GC-O-MS. The highest dilution in which the compound was detected was the flavor dilution (FD) factor of that compound [27]. This was serial dilutions (1:3, 1:9, 1:27, ……, 3^n^) to ready injection, the log_3_FD were 1, 2, 3, ……, n respectively. Compounds with higher log_3_FD were considered more critical.

### 3.7. Gas Chromatography-Olfactometry-Mass Spectrometry (GC-O-MS) Analysis

The analysis of odorants was performed on a GC 7890A coupled to a Triple Quad 7000B (both from Agilent, Palo Alto, CA, USA), and equipped with a Sniffer 9000 Olfactometer (Brechbühler, Switzerland). Separations of odorant compounds in GC were performed on DB-5ms UI (30 m × 0.25 mm × 0.25 µm, J & W Scientific, Folsom, CA, USA), and DB-WAX (30 m × 0.25 mm × 0.25 μm, J & W Scientific). The carrier gas was ultra-high purity helium with a flow rate of 1.2 mL/min. GC oven program was 40 °C for 3 min, ramped at 5 °C/min to 200 °C, and then ramped at 5 °C/min to 230 °C and held at 230 °C for 3 min. The temperatures of the injector and the GC/MS interface were 250 °C and 280 °C respectively. Electron-impact mass spectra were generated at 70 eV, with *m*/*z* scan range from 45 to 650 amu with the ion source temperature of 230 °C. Compounds were identified according to NIST 2.0 mass spectra libraries installed in the GC-MS equipment. GC-O was performed by three panelists who had the experience of sniffing odorants for two years and trained for sniffing the authentic standards for 5 days.

### 3.8. Identification and Quantification of Volatile Compounds

The chemical identification was based on the comparison of the mass spectrum, retention index and odor description with reference compounds, and some critical odorants were verified by comparison with authentic standard compounds. Mass spectra identification was based on the NIST 2.0 mass spectra libraries. The RI values and odor descriptions on DB-5ms UI and DB-WAX column with those of linear retention indices (LRIs) having the same/similar odor quality and RI, previously reported in database [28] and literature [29]. The maximum allowable deviation range of RI values was ±20. *n*-Alkanes (C_7_–C_22_) were analyzed under the same conditions to calculate LRIs:

LRI = 100N + 100n (t_Ra_ − t_RN_)/(t_R(N + n)_ − t_RN_) (1)

which was described by Dool and Krazt [30]. The volatile extraction methods of DHS and SAFE were also applied for odorants quantification. The internal standard, 2-methyl-3-heptanone at a concentration of 500 ng/g (500 ng of the internal standard/1 g of the sample), was added into the sample with the injection of 1 μL. The concentration for each odorant compound was calculated as follows:

C_i_ = C_IS_ A_i_/A_IS_ (2)

The abbreviations C_i_, C_IS_, A_i_ and A_IS_ represent the concentration of an odorant, concentration of internal standard, peak area of an odorant and peak area of internal standard on GC chromatogram respectively.

### 3.9. Odor Activity Value (OAV)

The evaluating methods of contributions in odorants were FD factors and odor activity value (OAV). In the method of OAV evaluation, the threshold of odorant was from other relevant literatures. The OAV for odorant compound was calculated as follows:

OAV = C_i_/T_i_ (3)

The abbreviations C_i_ and T_i_ represent the concentration and threshold of an odorant, respectively.

### 3.10. Statistical Analysis

Principal component analysis (PCA) was applied to the aroma-active compounds in Jinhua ham by DHS and SAFE. The relationship between hams at different fermentation times and cooking conditions were shown by principal component analysis based on the data from the tables of aroma-active compounds in Jinhua ham (Table 1 and Table 2). Principal components (PC) that explained a total variance greater than 80% were selected and the varimax rotation method was applied. The SPSS 17.0 software package was used for data analysis.

## 4. Conclusions

With the combination of different fermentation times and two odorant extraction methods, 81 aroma-active compounds were identified by GC-O-MS of Jinhua ham. Among them, acids, saturated aldehydes, benzene derivatives, esters and lactones were regarded as critical compound categories in Jinhua ham. With the increase of fermentation time, the type and content of the odorants increased significantly. Thirteen predominant odorants were identified in Jinhua ham by DHS-GC-O-MS, including trimethylamine, 3-methylbutanal, pentanal, dimethyl disulfide, hexanal, heptanal, D-limonene, 2-pentylfuran, octanal, 1-octen-3-ol, acetic acid, butanoic acid and 3-methylbutanoic acid. Nine key odorants including hexanal, octanal, 2,6-dimethyl-pyrazine, nonanal, butanoic acid, 3-methylbutanoic acid, (*E,E*)-2,4-decadienal, γ-nonalactone and *p*-cresol were identified in Jinhua ham by SAFE-GC-O-MS. Compounds identified as the common important odorants extracted by both SAFE-GC-O-MS and DHS-GC-O-MS methods were hexanal, octanal, butanoic acid and 3-methylbutanoic acid, forming the principal aroma profile of fermented, fatty and green notes of Jinhua ham.

By the using of aroma extraction and analysis methods such as SAFE, DHS, GC-O-MS, the odor-active compounds of different fermentation periods were identified, and the relationship between hams of different fermentation periods were analyzed by principal component analysis (PCA), so that to provide theoretical basis for the optimization of processing technology parameter and the more production of better score of ham grading. Further study on the aroma profile of Jinhua ham may be useful for product quality control of traditional Chinese dishes.

## Abbreviations

DHS: dynamic headspace sampling
SAFE: solvent-assisted flavor evaporation
P&T: purge-and-trap
SPME: solid phase microextraction
GC-O-MS: gas chromatography-olfactometry- mass spectrometry
LRI: linear retention indices
AEDA: aroma extract dilution analysis
DHDA: dynamic headspace dilution analysis
STD: standard deviation
TDSA: thermal desorption system autosampler
CIS: cold injection system
PCA: principal component analysis
